# Macrophages: Key Players in the Battle against Triple-Negative Breast Cancer

**DOI:** 10.3390/ijms251910781

**Published:** 2024-10-07

**Authors:** Irena Padzińska-Pruszyńska, Paulina Kucharzewska, Agata Matejuk, Małgorzata Górczak, Małgorzata Kubiak, Bartłomiej Taciak, Magdalena Król

**Affiliations:** 1 Center of Cellular Immunotherapies, Warsaw University of Life Sciences, 02-787 Warsaw, Poland; irena_pruszynska1@sggw.edu.pl (I.P.-P.); paulina_kucharzewska-siembieda@sggw.edu.pl (P.K.); malgorzata_gorczak@sggw.edu.pl (M.G.); malgorzata_kubiak1@sggw.edu.pl (M.K.); bartlomiej_taciak1@sggw.edu.pl (B.T.); 2 Department of Immunology, Collegium Medicum, University of Zielona Góra, 65-417 Zielona Góra, Poland; a.matejuk@inz.uz.zgora.pl

**Keywords:** triple-negative breast cancer, tumor-associated macrophages, targeted therapy

## Abstract

Triple-negative breast cancer (TNBC) is a challenging subtype of breast cancer characterized by the absence of estrogen and progesterone receptors and HER2 expression, leading to limited treatment options and a poorer prognosis. TNBC is particularly prevalent in premenopausal African-descent women and is associated with aggressive tumor behavior and higher metastatic potential. Tumor-associated macrophages (TAMs) are abundantly present within the TNBC microenvironment and play pivotal roles in promoting tumor growth, progression, and metastasis through various mechanisms, including immune suppression and enhancement of angiogenesis. This review provides an in-depth overview of TNBC, focusing on its epidemiology, its molecular characteristics, and the critical influence of TAMs. It discusses the pathological and molecular aspects that define TNBC’s aggressive nature and reviews current and emerging therapeutic strategies aimed at targeting these dynamics. Special attention is given to the role of TAMs, exploring their potential as therapeutic targets due to their significant impact on tumor behavior and patient outcomes. This review aims to highlight the complexities of the TNBC landscape and to present the innovative approaches that are currently being pursued to improve therapeutic efficacy and patient survival.

## 1. Introduction

Triple-negative breast cancer (TNBC) is a highly heterogeneous subtype of breast cancer defined by the absence of progesterone and estrogen receptors and the lack of overexpression or amplification of the human epidermal growth factor receptor 2 (*HER2*) gene. This absence of hormone receptors and HER2 makes TNBC distinct from other breast cancer subtypes, as it does not respond to hormonal therapy or therapies that target HER2 receptors, resulting in limited treatment options and a generally poorer prognosis [1].

Within the tumor microenvironment of TNBC, tumor-associated macrophages (TAMs) represent the most abundant immune cell population. Extensive research has demonstrated a significant association between TAM infiltration and the aggressive behavior of tumors, highlighting their crucial role in tumor growth, progression, and metastasis [2].

This review aims to provide a comprehensive overview of the current understanding of TNBC, covering its epidemiology, molecular and clinical characteristics, prevalence, biomarkers, and prognostic factors, as well as available therapeutic strategies. Additionally, it emphasizes the role of TAMs, exploring their impact on the development and progression of TNBC and their potential as targets for novel therapeutic approaches.

## 2. Concept of Breast Cancer

**Figure 1:**
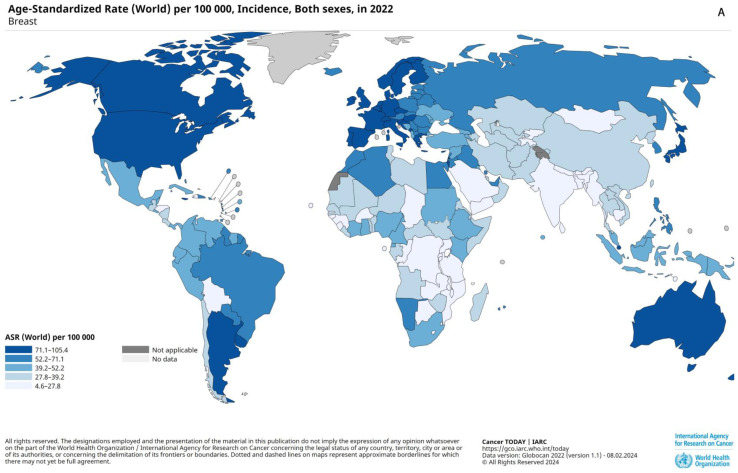

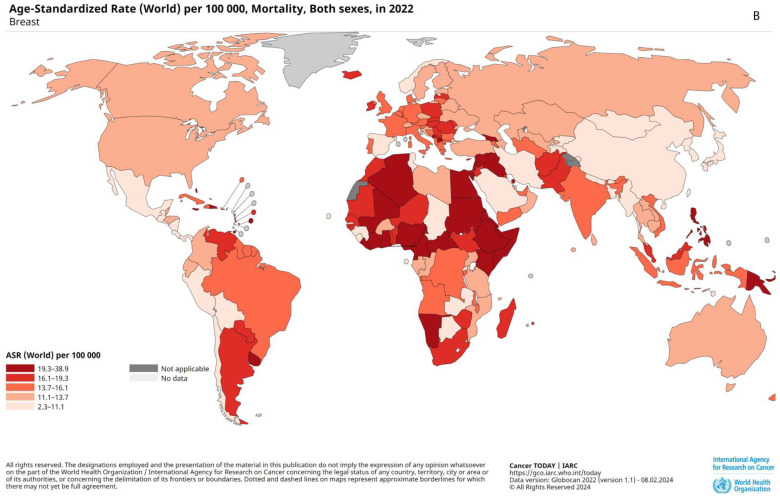
Global distribution of breast cancer incidence and mortality rates. (**A**) Age-standardized incidence rates (ASR) of breast cancer per 100,000 women worldwide. The highest incidence rates are observed in North America, Europe, and Australia, indicating a strong correlation with higher-income regions. (**B**) Age-standardized mortality rates (ASR) of breast cancer per 100,000 women worldwide. Higher mortality rates are prevalent in Africa, India, and South America, regions often associated with lower income and limited access to healthcare, highlighting significant global disparities in breast cancer outcomes [19].

Breast cancer is a major global health concern and the most frequently diagnosed cancer worldwide, accounting for approximately one in eight cancer cases [3,4]. Approximately 2.3 million new breast cancer cases were diagnosed in 2020 worldwide, which was a significant increase from the estimated 1 million cases in 2008 [5,6]. The incidence of breast cancer is rising globally, with higher rates in developed countries and greater mortality in less-developed regions [4]. In 2020 alone, it was responsible for one in six cancer-related deaths among women [7]. Male breast cancer is rare, with an incidence rate of about 1%, and is often diagnosed at a more advanced stage than in female patients [8]. There are conflicting reports on mortality in male breast cancer. Some studies indicate a higher mortality rate in male breast cancer, but other studies show the opposite results [8,9]. Breast cancer is commonly categorized into several subtypes: Luminal A (ER-positive with low histological grade), Luminal B (ER-positive with high histological grade), HER2-enriched, Claudin-low, basal-like (BL1 and BL2), immunomodulatory (IM), mesenchymal (M), mesenchymal stem-like (MSL), and normal breast-like tumors [10]. A graphical representation of the global distribution of breast cancer incidence and mortality rates is shown in Figure 1.

### 2.1. Pathological and Molecular Characterization of TNBC

TNBC is often characterized by aggressive behavior and higher mortality rates compared to other breast cancer subtypes [11]. Approximately 75% of TNBC cases belong to the basal-like category of breast cancers [12]. These tumors lack PR, ER, and HER2 and exhibit high expression of markers such as CK5, CK14, p53, p63, caveolin-1, and the epidermal growth factor receptor (EGFR) [12,13]. Alterations in the EGFR/PI3K/PTEN/Akt/mTORC1/GSK-3 signaling pathway due to mutations or the abnormal expression of pathway-related genes are frequently observed in TNBC [14]. Lehmann et al. used gene expression profiling to categorize TNBC into several heterogeneous subtypes, basal-like-1 (BL1), basal-like-2 (BL2), mesenchymal (M), mesenchymal stem-like (MSL), luminal androgen receptor (LAR), and immunomodulatory (IM) subtypes, which after further studies was refined from six to four groups (BL1, BL2, M, and LAR) [15,16]. Some researchers recognize the existence of an additional unstable subgroup (UNS) [17].

### 2.2. Prevalence, Epidemiology, and Risk Factors

TNBC accounts for approximately 15–20% of all breast cancer cases [18]. The incidence of TNBC is closely correlated with the overall breast cancer rates. Globally, breast cancer is most prevalent in regions such as North America, Europe, and Australia, where higher incidence rates are often associated with higher income levels and better healthcare infrastructure. However, when examining mortality rates, Africa, India, and South America are the regions most affected by TNBC due to lower income levels and limited access to advanced medical care. This disparity highlights the significant impact of socio-economic factors on breast cancer outcomes [19]. TNBC affects about 170,000 women annually, with the highest prevalence among African American and sub-Saharan African women, which contradicts trends in breast cancers overall [20].

American Indian/Alaska Native women are also disproportionately affected by this subtype [21]. Risk factors include mutations in the *BRCA1* or *BRCA2* genes, which are associated with a tenfold increase in the risk of developing breast cancer [22]. Germline mutations in *BRCA1* are present in 11–20% of TNBC cases, and TNBC accounts for 70% of breast cancers in *BRCA1* mutation carriers [23]. *BRCA1* mutations have been identified in 15.5% of TNBC cases diagnosed in women under 40 years of age [24].

Metabolic abnormalities, rather than body mass index (BMI), have been linked to the risk of TNBC [25]. Specific mechanisms connecting adiposity to TNBC in African American women have been proposed [26]. Additionally, an increased consumption of carbohydrates, particularly fructose, is also positively associated with TNBC risk [27]. Surprisingly, physical activity, while being protective against breast cancer in general, does not seem to significantly reduce TNBC risk due to its hormone-independent nature [28,29].

Reproductive factors such as younger age at first pregnancy or at menopause and older age at menarche are generally associated with a reduced risk of breast cancer [30,31]; however, these factors do not seem to influence TNBC risk [32]. Extended breastfeeding duration offers protective effects against TNBC, particularly in parity-associated breast cancer (PABC) [32,33]. Oral contraceptive use, particularly for more than five years or for over 15 years cumulatively, has been linked to an increased risk of TNBC [34], with a more substantial impact observed in women aged 20–39 [35].

Hormone replacement therapy (HRT) is associated with an increased incidence of breast tumors, particularly luminal subtypes, but studies have not consistently linked HRT to TNBC risk [36,37,38]. The biological mechanism of HRT is thought to promote the growth of existing carcinomas or induce new malignancies [39].

### 2.3. Prognostic Factors

Prognostic factors associated with reduced overall survival (OS) and disease-free survival (DFS) in TNBC include smoking, advanced clinical stage, larger tumor size, angiolymphatic and perineural invasion, positive sentinel lymph node, axillary node involvement, older age, higher cancer burden, and the presence of *PIK3CA* mutations [40,41,42,43]. Genetic tests such as the Breast Cancer Index and Mammostrat are approximately 62% and 65% accurate, respectively, varying based on population and disease stage [40,43].

### 2.4. Conventional Approaches in Diagnosis and Treatment

Breast cancer diagnosis typically involves clinical examination, imaging, and immunohistopathological analysis [44]. These assessments help determine cancer stage using the TNM classification system [45]. Mammography is utilized in about 60% of breast cancer diagnoses. Disparities in mammography screening rates, particularly among women over 66 years old, contribute to differences in breast cancer detection and outcomes, with African American women being less likely to have received screening [46]; however, this does not fully account for the higher incidence of TNBC among African American women [47].

TNBC, lacking ER, PR, and HER2 receptor expression, presents significant therapeutic challenges due to its aggressive nature and poor response to conventional therapies. Standard treatment includes neoadjuvant therapy, adjuvant therapy, surgery, and radiotherapy. Neoadjuvant therapy often involves doxorubicin and cyclophosphamide followed by paclitaxel, with or without cisplatin [48,49]. Alternative agents include carboplatin and bevacizumab [50]. In adjuvant settings, anthracyclines and taxanes are commonly used [51]. Chemoresistance remains a significant barrier to successful TNBC treatment, necessitating alternative strategies such as capecitabine, docetaxel, or ixabepilone [52,53,54].

### 2.5. Modern Approach to TNBC Treatment

The suboptimal outcomes associated with traditional treatments for TNBC, including surgery, radiotherapy, and chemotherapy, have driven ongoing research efforts to develop novel therapeutic strategies for this aggressive cancer subtype.

For patients with TNBC who carry *BRCA* mutations and exhibit resistance to conventional chemotherapy, alternative therapeutic options include platinum-based chemotherapeutic agents such as cisplatin or carboplatin [55], as well as targeted poly (ADP-ribose) polymerase (PARP) inhibitors like olaparib and talazoparib [56].

In advanced TNBC cases characterized by the expression of the programmed death-ligand 1 (PD-L1) protein, initial treatment protocols may involve the integration of immunotherapy (e.g., pembrolizumab) with chemotherapy. It is estimated that approximately 20% of TNBC cases exhibit PD-L1 expression [57]. Furthermore, pembrolizumab has demonstrated efficacy in TNBC cases presenting with high levels of microsatellite instability (MSI) or mutations in mismatch repair (MMR) genes [58]. Additionally, TNBC patients with a high tumor mutational burden (TMB-H) may benefit from pembrolizumab treatment [59]. A specific group of patients suffering from TNBC are HER-low patients. Thanks to the presence of the HER receptor, modern therapies using anti-HER2 agents can be used, which positively impacts patient outcomes. An example of such therapy is trastuzumab deruxtecan, an antibody directed against HER-2 combined with a topoisomerase inhibitor, causing DNA damage and the apoptosis of cancer cells [60]. Human trophoblastic cell surface antigen 2 (Trop-2) is a transmembrane calcium signal transducer that is highly expressed on the membrane surface of epithelial cells in various tumor types. While Trop-2 is overexpressed across all breast cancer subtypes, its levels are particularly elevated in triple-negative breast cancer (TNBC). In cases of advanced TNBCs that have been refractory to at least two prior therapeutic regimens, the anti-Trop-2 antibody–drug conjugate sacituzumab govitecan has emerged as a viable treatment option [61]. Targeting the PI3-kinase (PI3K) pathway in triple-negative breast cancer is a promising therapeutic strategy because this pathway is dysregulated in approximately 25% of TNBC patients, driving tumor growth, survival, and resistance to therapy [62]. Inhibiting PI3K can disrupt the critical signaling involved in cancer cell proliferation and survival, potentially slowing tumor progression. This group of drugs, exemplified by Alpelisib, a small-molecule α-selective PI3K inhibitor, is already being tested in clinical trials on patients with TNBC [63].

Nutritional science has increasingly focused on the exploration of dietary interventions and supplements as adjunctive therapies for cancer. Diets such as the fasting-mimicking diet, Mediterranean diet, and ketogenic diet are under investigation for their potential to positively impact TNBC outcomes [64,65,66]. Several studies have also examined the antineoplastic effects of various nutritional supplements, particularly omega-3 polyunsaturated fatty acids, vitamin D, selenium, and eicosapentaenoic acid/docosahexaenoic acid [67,68,69].

These advancements represent a multifaceted approach to the management of TNBC, incorporating targeted therapies, immunotherapy, and supportive dietary strategies to address the unique challenges posed by this breast cancer subtype.

## *3.* Tumor-Associated Macrophages in TNBC

Macrophages have multiple embryonic origins, including erythro-myeloid progenitors (EMPs) found in the yolk sac and fetal liver, as well as macrophage/dendritic cell progenitor cells (MDPs) located in the bone marrow, which produce monocytes. These macrophages have the ability to self-replicate, and their origins can shift over time as bone marrow-derived macrophages replace those initially originating from the yolk sac. During tumor development, monocytes from the bone marrow are attracted by chemotactic signals and subsequently differentiate into macrophages within the tumor environment [70].

According to traditional classification, macrophages are divided into two subtypes, M1 and M2 [71]. M1, by promoting inflammatory responses, possesses anti-cancer properties, while M2, by displaying inflammation-inhibitory features, promotes tumor growth and metastasis [72,73]. However, this binary model of TAMs in the tumor microenvironment (TME) has been challenged based on the new findings derived from novel single- and bulk-cell genomic studies that describe highly heterogeneous subpopulations of these cells in breast cancer tissues [74,75,76]. Nine macrophage “spectrum models” with pro- and anti-tumor properties have been described [73,77,78]. The phenotype of macrophages within TME in breast cancer depends on the cancer subtype, microanatomical location, and existing tumor microenvironment [79,80]. TNBC is characterized by a specific inflammatory microenvironment in which high levels of molecules released from activated inflammatory cells, such as pro-inflammatory cytokines, ROS (reactive oxygen species), and reactive nitrogen species (RNS) coexist with angiogenic factors, such as the Vascular Endothelial Growth Factor (VEGF), and with a significant number of Tumor-Infiltrating Lymphocytes (TILs) and TAMs [81]. As in many cancers, TNBC progression, growth, and tumor metastasis rely on communication and crosstalk between cells, creating a tumor environment and infiltrating cells [82]. Cellular communication is based on the release of biologically active factors that, in an autocrine and/or paracrine manner, directly influence tumor progression. Soluble factors such as cytokines (e.g., VEGF, IL-4, and IL-10), chemokines (CCL2, CCL5, CCL7, and CCL22), and enzymes like COX-2, IDO, and MMPs have a direct effect on the suppression of anti-cancer immune responses [83,84].

At the early stages of TNBC, infiltrating macrophages play a crucial role in anti-tumor immunity; however, with tumor progression, under the influence of TME, they start to nourish cancer cells and promote pro-tumorigenic activity [82,85]. Several mechanisms within the TME reprogram them to facilitate TNBC progression, invasion, and angiogenesis, which negatively impacts therapies and clinical outcomes. The negative effects of the TME in triple-negative breast cancer are based on the suppression of natural immune mechanisms, promotion of the invasion-transfer cascade, and promotion of relapse resistance [86]. Another reason why TAMs significantly contribute to the failure of therapeutic approaches is due to drug resistance [87]. A high infiltration rate of TAMs (CD68+, CD163+) in TNBC is associated with poor patient prognosis [88,89,90] and is associated with a higher risk of distant metastasis [91].

### 3.1. Crosstalk between Tumor-Associated Macrophages and Triple-Negative Breast Cancer

Examining the crosstalk between TNBC and TAMs requires various research models to capture their complex and dynamic interactions, as no single model can fully replicate the intricate tumor microenvironment and its multifaceted effects on tumor progression and immune responses. Preclinical studies commonly utilize co-cultures, conditioned media, organoids from TNBC cell lines, and mice with xenografts of human TNBC tumors [92,93,94]. Increased TAM recruitment and invasiveness of TNBC are associated with high hypoxia-inducible factor (HIF-1α) regulatory gene expression and colony-stimulating factor 1 (CSF-1) secretion [95]. HIF-1α is a key crosstalk signal between TAMs and the TME. It has been found that TNBC has increased the expression of genes regulated by HIF-1α [96]. A hypoxic state and HIF-1α molecule expression within the TME trigger pro-tumorigenic Treg cell recruitment via the upregulation of CCL20 cytokines [97]. Additionally, hypoxia contributes to angiogenesis and further immune suppression by inhibiting NK cell and cytotoxic T lymphocyte responses [98]. The production of angiogenic growth factors and the mobilization of bone marrow-derived angiogenic cells are also a consequence of HIF-1α activity. TAMs promote angiogenesis and metastasis through extracellular matrix reconstruction by producing proteolytic enzymes and matrix metalloproteinases [99].

**Figure 2:**
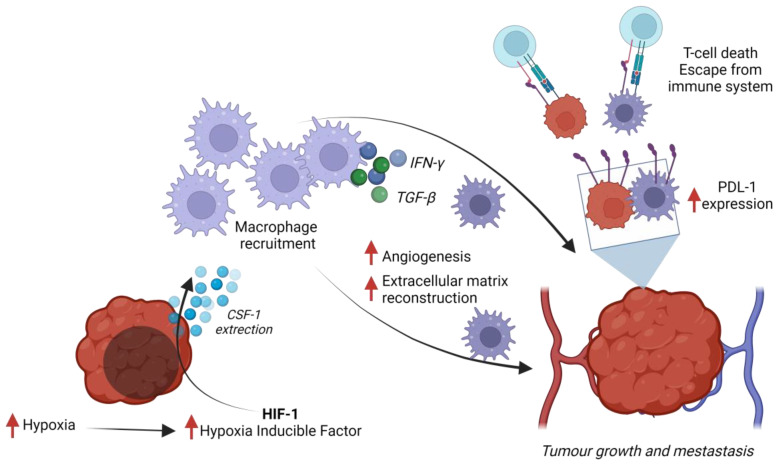
Crosstalk between TAMs and TNBC: key mechanisms in the tumor microenvironment. This diagram illustrates the complex interplay within the tumor microenvironment of TNBC, highlighting the interactions among immune cells, signaling molecules, and hypoxic conditions that drive tumor progression and metastasis. Central to this depiction are TAMs, whose recruitment is triggered by tumor cells’ increased secretion of CSF-1. This secretion is enhanced under the low-oxygen conditions that upregulate HIF-1α. This diagram also details how TAMs contribute to the modulation of immune responses through PD-L1 expression, resulting in T-cell apoptosis and immune system evasion. Additionally, it shows how these recruited macrophages support tumor growth and spread by promoting angiogenesis and reconstructing the extracellular matrix.

Acriflavine and other HIF-1α inhibitors, digoxin, as shown by Chaturvedi et al., blocked the signaling and recruitment of TAMs and MDSCs (myeloid-derived suppressor cells, TAM progenitors), suggesting that the addition of HIF-1α inhibitors to existing therapeutic regimens may improve the clinical outcome in patients with TNBC [96]. The activation of lymphatic endothelial cells and the formation of new blood vessels are also consequences of the upregulation of b4 integrin and TGF-b1 by TAMs activated by the TME [100]. Additionally, TGF-bs, multifunctional, pleiotropic molecules with key roles in cell differentiation, proliferation and migration, extracellular matrix metabolism, and immunosuppression [101], contribute to PD-1+ Treg accumulation, which suppresses effector T cells and plays a role in TNBC immune escape. TNBC cells highly express PD-L1, a ligand for programmed death receptor 1 (PD-1) that strongly inhibits the effectiveness of T-cell killing [102,103] and can be regulated by TAMs via the JAK/STAT3 and PI3K/AKT signaling pathways and IFN-γ production [104]. TNBC secretes cytokine CSF-1, which binds to the CSF-1 receptor (CSF-1R) on TAMs and stromal cells like mesenchymal stem cells (MSCs) that drive their recruitment to the primary tumor and increase metastasis to the lymph nodes and lungs [96]. TNBC, compared to other BC types, secretes more Granulocyte-Colony Stimulating Factor (G-CSF), which promotes the conversion of M1 to M2 macrophages [102]. IL-4 secretion from TNBC cells, regulated by HUNK (hormonally upregulated neu-associated) kinase activity, drives the polarization of macrophages into an M2-like phenotype. In turn, IL-4 promotes cancer metastasis and stimulates macrophages to release epidermal growth factor. This outlines a paracrine signaling loop between tumor cells and TAMs, governed by HUNK and mediated through IL-4/IL-4 receptor interactions, and targeting HUNK could be a TAM-altering strategy [105]. In vitro studies on the interaction of TNBC cells with monocytes have shown that cytokines secreted by cancer cells cause the transformation of monocytes into macrophages with mixed M1 and M2 characteristics, but with a pro-tumor effect characteristic of M2 cells [94]. Besides the previously mentioned cytokines, several others can be released by TAMs in TNBC that directly or indirectly affect tumor-promoting and immunosuppressive abilities. The interaction between NLRP3 inflammasomes, inflammatory cytokines like IL-18 and IL-1b, and TNBC has been found to promote cancer development with the potential for novel anti-inflammasome treatments in this type of cancer [106]. Co-cultured TNBC cells with macrophages showed an increased ability to metastasize and higher viability via ROS elevation and IL-1α expression [92]. The close crosstalk between TAMs and the TME is evident at every stage of cancer development, progression, and metastasis. Since there are currently no highly satisfactory therapeutics for TNBC, scientists are working on promising novel therapies focusing on TAM targeting, which cover the elimination, inhibition, and reprogramming of these cells [107,108]. A graphical summary of the relationship between TAMs and TNBC is shown in Figure 2.

### 3.2. Regulation of PD-1 Expression by TAMs

Macrophages regulate PD-L1 expression in tumor cells and other TAMs via multiple signaling pathways. Transforming growth factor-beta (TGF-β) enhances PD-L1 expression through the AKT/NF-kB or AKT/β-catenin pathway. Interferon-gamma (IFN-γ) promotes PD-L1 transcription by activating STAT1, while prostaglandin E2 (PGE2) increases PD-L1 expression through the PI3K/Akt/mTOR pathway, often in response to pro-inflammatory cytokines. Osteopontin (OPN) and interleukins such as IL-1a, IL-10, and IL-27 also contribute to PD-L1 upregulation through various pathways, including NF-kB/p65 and STAT1 signaling [109].

The impact of PD-1/PD-L1 signaling on macrophages includes altered macrophage function and polarization. PD-1+ TAMs exhibit reduced phagocytic activity and co-stimulatory molecule expression, contributing to an immunosuppressive TME. PD-1 also negatively regulates macrophage survival pathways, leading to increased apoptosis [110]. Various stimuli can increase PD-1 expression in macrophages. Elevated PD-1 levels inhibit key signaling pathways, such as the Janus N-terminal kinase (JNK) and PI3K/Akt pathways, by recruiting SHP-2. This suppression impairs macrophage function and reduces the expression of co-stimulatory molecules like CD86, as well as MHC I and II proteins. Research indicates that PD-1+ TAMs exhibit diminished phagocytic activity compared to their PD-1-counterparts. Additionally, PD-L1 exosomes released by tumor cells reinforce PD-L1 expression in macrophages, promoting their polarization toward an M2 phenotype [111].

### 3.3. Targeting Tumor-Associated Macrophages in Triple-Negative Breast Cancer

Clinical studies have demonstrated a significant association between TAMs and increased mortality rates in cancer patients [112]. In TNBC, the TME is particularly effective at producing cytokines that gradually reprogram macrophages, promoting tumor invasion and metastasis [113]. Because of the crucial role that TAMs play in the progression of TNBC, targeting these cells has emerged as a promising strategy for chemo-immunotherapy to inhibit tumor growth. This section explores several novel approaches for targeting the immunosuppressive TME in TNBC. As detailed in Table 1, the ongoing clinical trials evaluate a range of innovative approaches, from immune checkpoint inhibitors to targeted therapies, to refine treatment regimens and address resistance mechanisms.

#### 3.3.1. Inhibition of Macrophage Recruitment to the Tumor

Reducing the recruitment of monocytes into the TME is a key strategy to limit macrophage infiltration in breast cancer. This can be achieved by blocking the CCL2/CCR2 and CSF-1/CSF-1R pathways and other chemoattractants such as M-CSF, VEGF, CXCL-12, and CCL5. The overexpression of CCL2 in breast tumors has been linked to increased macrophage infiltration, enhanced cancer metastasis, and poor prognosis [114]. Additionally, CCL2 promotes TAMs’ secretion of IL-1β, which further contributes to TNBC metastasis [115]. The inhibition of CCL2 has been shown to reduce M2 macrophage recruitment in TNBC models [116].

Carlumab, a human monoclonal antibody targeting CCL2, has been effective in depleting macrophages from tumors and reducing disease recurrence following chemotherapy [117]. Other agents, such as Bindarit and trabectedin, inhibit CCL2 synthesis, and as a result, they prevent macrophage infiltration into tumors. Bindarit, a synthetic indazole derivative, has been shown to reduce TAM infiltration in a breast cancer animal model by blocking monocyte chemotactic proteins CCL2, CCL7, and CCL8 [118,119,120]. Trabectedin, a tetrahydroisoquinoline alkaloid, alters the TME by suppressing monocyte recruitment and preventing their differentiation into TAMs. This compound disrupts the cell cycle by binding to DNA and has been shown to reduce the expression of cytokines like CCL2 and IL-6, which are involved in angiogenesis and inflammation [121]. Although a phase II trial of trabectedin in metastatic TNBC showed limited response, partial responses were observed in cases of HER2-overexpressing metastatic TNBC [122]. Blocking the CCL2 receptor CCR2 (C-C motif chemokine receptor 2) is another approach to reduce macrophage recruitment and their further activity.

CSF-1, also known as macrophage colony-stimulating factor (M-CSF), and its receptor CSF-1R are crucial for the differentiation, migration, and survival of macrophages and monocytes. Inhibiting the CSF-1 pathway can decrease TAM populations within tumors. High levels of CSF-1 expression in TNBC are associated with higher tumor grades [123]. Lacnotuzumab (MSC110), a monoclonal antibody against CSF-1, has demonstrated anti-tumor efficacy in combination with spartalizumab in several tumor types, including TNBC, in a phase Ib/II study [124]. However, a phase II study combining lacnotuzumab with carboplatin and gemcitabine in advanced TNBC showed anti-tumor activity comparable to chemotherapy alone [125]. Clinical trials based on small molecules/mAbs targeting the CSF-1/CSF-1R pathway for solid tumors, including TNBC, are ongoing [126]. The results from clinical phase III trials show that in advanced TNBC, the CSF-1R inhibitors like PLX3397, LY3022855, and cabiralizumab, although with positive effects on CD8+ cell function, eventually develop resistance [127].

Interleukin-6 (IL-6) is another cytokine that promotes the growth and survival of TNBC, facilitating anchorage-independent colony formation and resistance to apoptosis [128]. High levels of IL-6 in breast cancer patients have been linked to a worse prognosis [129]. Siltuximab, a monoclonal antibody that targets IL-6, reduces plasma levels of several chemoattractants, including CCL2, VEGF, and CXCL-12, potentially inhibiting TAM recruitment and function [130].

#### 3.3.2. Direct Elimination of TAMs

Recognizing the harmful impact of TAMs on cancer progression has led to the development of strategies to target and eliminate these cells directly. Several agents induce macrophage apoptosis or activate the immune system to target TAMs selectively.

Bisphosphonates, such as clodronate, selectively kill macrophages, thereby inhibiting tumor growth, angiogenesis, and metastasis [131]. Clodronate has also shown moderate activity against TNBC cells [132], with clinical trials confirming its efficacy in breast cancer treatment [133,134]. Zoledronic acid, another bisphosphonate, has proven effective in depleting TAMs, inhibiting angiogenesis and metastasis, and preventing macrophage differentiation into TAMs [135,136,137]. This agent selectively targets macrophages expressing MMP9, further enhancing its anti-cancer effects [138].

#### 3.3.3. Reprogramming TAMs into Anti-Tumor Macrophages

Modulating macrophage polarization presents a promising therapeutic strategy. It can be carried out either through the introduction of Th1-cytokines or by disrupting the transcriptional pathways that lead to M2 macrophage differentiation. Additionally, reprogramming macrophage function can be achieved by the modulation of the Toll-like receptor (TLR)/nuclear factor—κB (κB/NLRP)/Nod-like receptor pyrin domain-containing (NLRP) signaling pathway [139,140]. In triple-negative breast cancer studies, the delivery of TLR-3 and TLR-7 agonists induced macrophage polarization toward M1 [141].

An alternative approach involves enhancing the immune response by activating co-stimulatory molecules, such as CD40. A phase 1 study is currently underway to evaluate the efficacy and safety of a combinatorial approach involving chemotherapy, a CD40 agonist, and an Flt3 ligand in patients with metastatic triple-negative breast cancer (TNBC) [142]. Tasquinimod, an S100A9 inhibitor, has been shown to enhance the presence of M1 macrophages within the tumor microenvironment while simultaneously reducing neo-vascularization and monocyte infiltration [143]. Its potential therapeutic effects in breast cancer have been validated through both in vitro studies and in vivo experiments using murine models [144].

Mutations within the PI3K/Akt/mTOR pathway are frequently observed in certain subtypes of TNBC. PI3K inhibitors not only regulate cellular metabolism, growth, and survival but also promote the induction of the M1-like phenotype in tumor-associated macrophages [145]. Alpelisib, a PI3Kα-specific inhibitor, was already tested in clinical trials in TNBC patients with moderate positive effects and good tolerance [63,146].

As mentioned above, PD-1 is an inhibitory co-receptor that interacts with its ligand, programmed death ligand-1 (PD-L1), to downregulate T-cell activity [147]. The αPD-L1 treatment effectively inhibits the polarization of TAMs into the M2 subtype induced by IL-13 in vitro. This inhibition blocks the epithelial–mesenchymal transition (EMT) and stemness of TNBC cells and reduces their migration and angiogenesis. αPD-L1 achieves this by reversing TAM/M2 polarization and preventing the phosphorylation and nuclear translocation of STAT3. In vivo experiments showed that αPD-L1 reduced lung metastases without affecting tumor growth and decreased the expression of markers related to TAM/M2, EMT, stemness, and angiogenesis in tumor tissues. These findings highlight αPD-L1’s critical role in combating TNBC metastasis and angiogenesis, suggesting it could be a novel therapeutic strategy for treating clinically resistant TNBC [148]. Among the checkpoint inhibitors, pembrolizumab is an unquestioned leader. It demonstrated greater effectiveness in early-stage TNBC than a placebo, regardless of PD-L1 status. In advanced-stage TNBC, pembrolizumab proved as effective as single-agent chemotherapy but with a superior safety profile. Additionally, pembrolizumab combined with chemotherapy resulted in significantly improved median progression-free survival compared to chemotherapy alone in advanced TNBC cases [149]. The KEYNOTE-522 trial was the first phase 3 trial to assess pembrolizumab in early-stage TNBC within both neoadjuvant and adjuvant settings. Conducted from March 2017 to September 2018, the trial randomized 1174 patients to receive either 200 mg of pembrolizumab or a placebo every three weeks alongside standard chemotherapy regimens. The primary endpoints were pathological complete response (pCR) and event-free survival (EFS), with secondary endpoints focusing on pCR and EFS in the PD-L1-positive subgroup. Interim analyses showed that pembrolizumab significantly improved pCR rates by 13.6 percentage points and EFS by 7.7 percentage points at 36 months. These results led to FDA and EMA approval of pembrolizumab combined with chemotherapy as a neoadjuvant treatment, followed by adjuvant pembrolizumab for high-risk early stage TNBC [150,151,152,153,154].

Another strategy is the delivery of TAM-reprogramming molecules into M2 macrophages to change them into the M1 (tumor-fighting) phenotype. Several strategies have been developed to induce this phenotypic shift, including the use of specific inhibitors and modulators. For example, as mentioned before, pexidartinib, a CSF-1R antagonist, reduces M2 polarization and enhances the infiltration of CD8+ T cells, potentially overcoming the resistance to PD-1/PD-L1 inhibitors. Similarly, AZD5153, a BRD4 inhibitor, promotes M1 polarization and boosts anti-PD-L1 therapy efficacy by enhancing pro-inflammatory cytokine production [155]. Additionally, the inhibition of M2 polarization can be achieved in breast cancer by the usage of metformin, which decreases the proliferation of the cells and tumor size in triple-negative breast cancer [156,157,158].

The selective TAM-targeting-for-reprogramming process is challenging; however, this obstacle can be overcome by further drug delivery optimization using nanomaterials. For instance, the polymer *N*-(2-hydroxypropyl) methacryloyl (HMPA) demonstrates preferential localization within M2 macrophages, thereby enhancing the possibility of the targeted reprogramming of these cells [159].

One recent study presents a more comprehensive approach to the development of new treatment methods for TNBC based on the modification of TAM phenotype. The authors explore the potential of using a quantitative system pharmacology (QSP) model to investigate the dynamics of TAMs in TNBC and their response to various immunotherapeutic strategies. The computational model they developed simulates the interactions between TAMs, cancer cells, and therapeutic agents, allowing for the prediction of macrophage behavior under different immunotherapy conditions. Conducting this type of in silico clinical trial would provide insights into how manipulating TAM polarization could impact treatment efficacy before initiating actual trials. However, QSP model-based prediction has its limitations, as it simplifies the complex and dynamic interactions within TME, overlooks cancer heterogeneity, and relies on specific assumptions about cellular interactions and behavior. Furthermore, the accuracy of these simulations depends on the availability and quality of preclinical data. Incomplete or biased information can potentially lead to inaccurate predictions and misleading conclusions. To overcome these obstacles and improve the predictive capability of QSP model-based in silico trials, it is essential to adopt several strategies. For instance, the continuous addition of new experimental data can enhance model accuracy. Moreover, incorporating information about other immune cells and their interactions with TAMs and TNBC will provide a more comprehensive view of TAMs. Lastly, utilizing advanced statistical techniques for parameterization can help account for variability and uncertainty, leading to more reliable predictions [160].

#### 3.3.4. Activation of Macrophage Phagocytosis

Cells have mechanisms to evade phagocytosis by phagocytic cells, such as macrophages, by expressing anti-phagocytic molecules, commonly called phagocytic checkpoints. Notably, many cancer cells, including those in breast cancer, can express these phagocytic checkpoints, thereby evading detection and phagocytosis by M1 macrophages, which impairs their ability to mount an effective immune response [161]. One extensively studied phagocytic “don’t eat me” molecule in breast cancer is CD47. In TNBC mouse models, an anti-CD47 antibody conjugated with the cytotoxic drug mertansine demonstrated significant inhibition of tumor growth compared to the administration of the drug alone (Si et al., 2021). Other approaches include Evorpacept, a CD47-SIRPα inhibitor that improves macrophage phagocytosis and M1 polarization [155].

Anti-CD47 antibodies, such as Hu5F9-G4, have shown promise in targeting cancer cells, but their widespread expression in normal cells can lead to off-target effects like anemia, thrombocytopenia, and leukopenia. To mitigate anemia risk, a low-dose priming strategy with Hu5F9-G4 combined with rituximab has been proposed to target aged red blood cells and stimulate compensatory hematopoiesis selectively. The broad expression of CD47 also presents a challenge, potentially creating an “antigen sink” that necessitates higher doses or more frequent administration for an effective blockade. Researchers are developing safer strategies to address these issues, including identifying tumor-specific CD47 epitopes and creating bispecific antibodies [162].

### 3.4. Potential Challenges of TAM-Targeted Therapies

As TAM-targeting therapies become more prominent in the treatment of TNBC, several risks and challenges may arise. Understanding these potential limitations is crucial for developing more effective therapies. TAMs play an important role in the tumor immune microenvironment and are vital for maintaining homeostasis. For example, the non-selective and uncontrolled depletion of TAMs could disrupt the broader immune ecosystem and lead to a compromised immune response [163]. On top of that, non-specific TAM-targeted therapies can affect tissue-resident macrophages that are unrelated to cancer [164,165].

Another important issue to consider is the long-term implications of macrophage-targeted therapies. For instance, as previously mentioned, the inhibition of CCL2 effectively reduces M2 macrophage recruitment in TNBC models. However, studies have shown that interrupting a treatment with a CCL2-targeting agent in mouse tumor models leads to the accumulation of monocytes and the promotion of cancer growth, which highlights the importance of prolonged drug administration [166].

Furthermore, TAM targeting alone fails to generate a robust anti-tumor response, as cancer cells can activate compensatory mechanisms that allow them to mitigate therapeutic effects and develop drug resistance [167]. Several studies have shown that the prolonged inhibition of CSF-1R can result in acquired resistance and consequently tumor relapse via activation of the PI3K pathway. However, the co-administration of PI3K inhibitors with CSF-1R blockers has been shown to extend survival in preclinical studies [168]. This compensatory effect and accelerated tumor growth in response to prolonged CSF-1R inhibition have recently been linked to an augmented recruitment of granulocytes into the tumor microenvironment (TME). Kumar et al. found that the combination of CSF-1R inhibition and CXCR2 antagonists effectively prevents granulocyte infiltration, leading to enhanced anti-tumor responses [169]. Therefore, it is necessary to explore these compensatory responses and administer appropriate combined therapies at the right time to enhance treatment efficacy.

Furthermore, TAM targeting alone fails to generate a robust anti-tumor response, as cancer cells can activate compensatory mechanisms that allow them to mitigate therapeutic effects and develop drug resistance. Therefore, it is necessary to explore these compensatory responses and administer appropriate therapies at the right time to enhance treatment efficacy.

Additionally, according to the traditional macrophage polarization model, TAMs are classified as either an anti-tumor phenotype (M1) or a pro-tumor phenotype (M2). However, in recent years, this approach has evolved into a “spectrum model”, revealing more than just two activation states [170]. Thus, a more refined classification of TAMs, distinguishing between those with anti-tumor and pro-tumor roles, could facilitate more effective therapeutic interventions.

Overall, considering the diversity of TAMs and the potential development of resistance mechanisms caused by TAM-targeting drugs, it is essential to gain a deeper insight into how these agents function. This understanding is crucial for optimizing therapeutic efficacy and minimizing risks, whether they are used as a monotherapy or in combination with other therapeutic agents.

### 3.5. Future Perspectives: Clinical Trials Targeting Immune Checkpoints and Other Strategies in TNBC

TNBC is a particularly aggressive and heterogeneous subtype of breast cancer, characterized by its limited treatment options and high mortality rates. Recent advances in understanding TNBC biology have highlighted the pivotal role of TAMs in influencing cancer progression, immune evasion, and therapeutic resistance. TAMs contribute to TNBC’s malignancy by promoting angiogenesis, facilitating tumor cell migration, and suppressing anti-tumor immunity. The diverse nature of macrophages, shaped by their origin and tissue environment, further complicates their interactions with cancer cells and immune components, making it essential to understand these dynamics throughout tumor progression and treatment.

Emerging therapeutic strategies targeting TAMs in TNBC are being actively explored. These approaches include inhibiting TAM recruitment, depleting TAM populations, and blocking their pro-tumoral activities. While promising, these strategies face challenges such as a lack of specificity in broad macrophage depletion, which can lead to significant toxicity. Targeted therapies, such as those directed against sialic acid-binding immunoglobulin-type lectin (Siglec)-1 and drug delivery systems like encapsulated zoledronic acid, offer potential solutions to enhance therapeutic specificity and efficacy. Furthermore, strategies involving transient TAM depletion followed by recovery phases may help balance anti-tumor immune responses and minimize pro-tumoral effects. Innovative immunotherapeutic approaches are also on the horizon.

Recent years have seen a significant surge in research and development in cancer precision medicine, which refers to the treatment tailored to a specific subset of patients whose tumors exhibit distinct molecular or cellular characteristics, such as particular genomic alterations or expression patterns of genes and proteins [171]. This approach allows for more targeted and effective cancer treatment, minimizing harm to healthy tissues and improving patient outcomes. Molecular profiling can also be applied in the stratification of patients for the development of more effective and precise therapies for TNBC. This strategy is especially important given the heterogeneity of this subtype of breast cancer and the limited effective therapeutic options currently available [172]. As previously mentioned, molecular subtyping of TNBC through gene expression profiling has identified distinct subgroups, such as basal-like and mesenchymal subtypes, which exhibit different levels of TAM infiltration and immune profiles. For instance, mesenchymal TNBC tends to have higher TAM infiltration and may benefit more from TAM-targeted therapies [173,174]. Moreover, advanced proteomic and metabolomic approaches could also be used to profile the immune landscape in TNBC, including TAM activity and polarization. These methods may help in the future to identify biomarkers related to TAM function, providing insights into how TAM-targeted therapies might be adjusted to specific TNBC subtypes. However, despite these clear advantages, the development of these biomarker-based TAM-targeted therapies is still in early stages, and to the best of our knowledge, there are not yet many examples of this approach being applied in practice.

In summary, the intricate role of TAMs in TNBC underscores their potential as therapeutic targets. Integrating TAM-directed therapies with existing treatments, including chemotherapy and immunotherapy, may offer synergistic benefits, improving anti-tumor immunity and patient outcomes. Continued research into TAM biology and novel therapeutic strategies is crucial for advancing TNBC treatment and improving patient outcomes in this challenging cancer subtype.

## Figures and Tables

**Table 1:** A list of the potential therapeutic candidates for TAM-directed therapies for TNBC with examples of clinical trials.

Drugs for Inhibition of Macrophage Recruitment to the Tumor				
Drug	Mechanism of Action	Other Drugs/Interventions Used in Trial	ClinicalTrials.gov ID	Phase
Trabectedin	Alkylating agent suppressing monocyte recruitment	Dexamethasone	NCT00580112	Phase II; completed
		Dexamethasone	NCT00050427	Phase II; completed
Lacnotuzumab (MSC110)	Monoclonal antibody against CSF-1	PDR001	NCT02807844	Phase Ib/II; completed
		Carboplatin, gemcitabine	NCT02435680	Phase II; completed
PLX3397	CSF-1/CSF-1R inhibitor	Eribulin	NCT01596751	Phase Ib/II; completed
		Multiple drugs	NCT01042379	Phase II; recruiting
LY3022855	CSF-1/CSF-1R inhibitor	-	NCT02265536	Phase I; completed
Cabiralizumab	CSF-1/CSF-1R inhibitor	Nivolumab, carboplatin, paclitaxel	NCT04331067	Phase Ib/II; active, not recruiting
**Drugs for direct elimination of TAMs**				
**Drug**	**Mechanism of action**	**Other drugs/interventions used in trial**	**ClinicalTrials.gov ID**	**Phase**
Clodronate	Bisphosphonate selectively killing macrophages.	Placebo	NCT00009945	Phase III; completed
		Ibandronate, zoledronic acid	NCT00127205	Phase III; completed
Zoledronic acid	Bisphosphonate depleting TAMs and inhibiting angiogenesis	Atorvastatin, standard neoadjuvant chemotherapy	NCT03358017	Phase II; completed
		-	NCT04045522	Unknown status
		-	NCT02595138	Phase III; unknown status
**Drugs for reprogramming TAMs into anti-tumor macrophages**				
**Drug**	**Mechanism of action**	**Other drugs/interventions used in trial**	**ClinicalTrials.gov ID**	**Phase**
Alpelisib	PI3Kα-specific inhibitor promoting M1-like phenotype	Nab-paclitaxel	NCT04251533	Phase III; active, not recruiting
		Nab-paclitaxel, iNOS inhibitor	NCT05660083	Phase II; recruiting
		Enzalutamide	NCT03207529	Phase I; completed
Pembrolizumab	PD-1 inhibitor used in combination therapy	Lenvatinib	NCT04427293	Phase I; recruiting
		Intraoperative radiation therapy	NCT02977468	Phase I; recruiting
		Olinvacimab	NCT04986852	Phase II; recruiting
Capivasertib (AZD5153)	BRD4 inhibitor promoting M1 polarization	Fulvestrant	NCT01226316	Phase I; active, not recruiting
		Paclitaxel	NCT02423603	Phase II; active, not recruiting
		Enzalutamide, Fulvestrant	NCT03310541	Phase I; completed
Metformin	Agent used to inhibit M2 polarization and decrease tumor size	Night fasting	NCT05023967	Phase IIb; recruiting
		Doxycycline	NCT02874430	Phase II; active, not recruiting
		Calorie restriction	NCT04248998	Phase II; active, not recruiting
**Drugs for activation of macrophage phagocytosis**				
**Drug**	**Mechanism of action**	**Other drugs/interventions used in trial**	**ClinicalTrials.gov ID**	**Phase and stage**
IMM2520	Anti-CD47 and PD-L1 bispecific antibody	-	NCT05780307	Phase I; recruiting
Evorpacept (ALX148)	CD47-SIRPα inhibitor improving macrophage phagocytosis	Fam-Trastuzumab Deruxtecan-Nxki	NCT05868226	Phase I/Ib; recruiting
Hu5F9-G4	Anti-CD47 antibody for targeting cancer cells	Olaparib	NCT05807126	Phase I; withdrawn

## Data Availability

Not applicable.
